# Characteristics for gait parameters of community-dwelling elderly Japanese with lower cognitive function

**DOI:** 10.1371/journal.pone.0212646

**Published:** 2019-03-27

**Authors:** Yu Taniguchi, Yutaka Watanabe, Yosuke Osuka, Akihiko Kitamura, Satoshi Seino, Hunkyung Kim, Hisashi Kawai, Ryota Sakurai, Hiroki Inagaki, Shuichi Awata, Shoji Shinkai

**Affiliations:** 1 Research Team for Social Participation and Community Health, Tokyo Metropolitan Institute of Gerontology, Tokyo, Japan; 2 Research Team for Promoting Independence of the Elderly, Tokyo Metropolitan Institute of Gerontology, Tokyo, Japan; 3 Research Team for Human Care, Tokyo Metropolitan Institute of Gerontology, Tokyo, Japan; 4 Research on Social and Human Science, Tokyo Metropolitan Institute of Gerontology, Tokyo, Japan; University of Bern, SWITZERLAND

## Abstract

**Objectives:**

Recent studies reported that several gait parameters were associated with lower cognitive function or cognitive decline, however, known gait parameters were limited and no study has used large-scale data. We identified the characteristics for gait parameters of community-dwelling elderly Japanese with lower cognitive function.

**Methods:**

1,240 community-dwelling adults (mean [SD] age, 77.2 [4.8] years; women, 59.4%) aged 70 or older participated in geriatric health assessments in 2016. We measured comprehensive gait parameters using resistive pressure platform. Cognition was assessed by Mini-Mental State Examination (MMSE).

**Results:**

There are possible correlations between gait measures (gait speed, stride length, step length, step width, average foot pressure, double support duration, and single support duration) and CVs (CV of stride length, step length, average foot pressure, and single support duration) with MMSE score, respectively. After adjustment for important confounders, multiple regression models showed that gait speed (β = .080, *p* = 0.006), stride length (β = .123, *p*<0.001), step length (β = .123, *p*<0.001), average foot pressure (β = .060, *p* = 0.040), double support duration (β = -.082, *p* = 0.004), single support duration (β = .086, *p* = 0.003), CV of stride length (β = -.091, *p*<0.001), CV of step length (β = -.090, *p*<0.001), and CV of single support duration (β = -.058, *p* = 0.037) had significant association with MMSE score, respectively.

**Conclusions:**

Our findings suggest that person with lower cognitive function tend to have unsteady gait such as erratic length and time of one step, in addition to decreasing the vertical displacement of the center of gravity and slower speed.

## Introduction

Patients with dementia, especially Alzheimer’s disease (AD) would develop cognitive dysfunction before the onset of dementia. The prodromal stage of dementia is defined as Mild Cognitive Impairment (MCI), Mild Cognitive Disorder, and/or Aging-Associated Cognitive Decline etc. Manly et al. reported that 21.8% of MCI were subsequently diagnosed with AD, however 47% of MCI remained unchanged and 31% reverted to normal over 10,517 person years [1]. Prevention or delay of dementia might be feasible for older persons in the prodromal stage of dementia, in addition to cognitively normal.

Verghese et al. newly described Motoric Cognitive Risk Syndrome (MCR), which shows predementia syndrome characterized by the presence of slow gait and cognitive complaints in older individuals without dementia or mobility disability [2]. MCR is a predictor of major cognitive decline and dementia [2, 3]. Montero-Odasso et al. reported that slow gait and cognitive impairment combined showed a higher risk for incident dementia among non-demented older adults [4]. Previous studies suggested that elderly in the prodromal stage of dementia might occur both motor and cognitive function deteriorate.

Earlier studies showed the associations between gait parameters and cognitive function and cognitive decline among community-dwelling older adults [5–8]. The cross-sectional study reported the association of poor performance on most absolute gait measures with executive function, attention and processing speed [9]. In our previous longitudinal study, we compared the association of component of gait speed (ie, step length or frequency) with cognitive decline among community-dwelling older Japanese, and concluded that step length is a better predictor than gait speed for cognitive decline [10]. Verghese et al. examined the relationship of quantitative gait factors with decline in cognitive domains in a population of non-demented older adults, and concluded that only pace factor was associated with global cognitive decline [11].

Recent studies reported that several gait parameters were associated with cognitive function or cognitive decline, however, known gait parameters especially age-related gait factors (i.e. step width [12], foot pressure and coefficient of variations (CVs) [13]) were limited, and no study has used large-scale data to identify comprehensive characteristics for gait parameter among older adults with lower cognitive function. Identifying the characteristic for gait parameters of elderly with lower cognition might yield new insights regarding physiological mechanism in the prodromal stage of dementia and facilitate clinical screening of high-risk persons. Therefore, our objective was to identify the gait parameters that strongly associated with lower cognition with using large sample of community-dwelling older Japanese.

## Methods

### Participants

Data for this study were collected as part of a community-wide survey in Takashimadaira, Itabashi ward, Tokyo in 2016. We had mailed to 7,614 adults aged 70 years or older who was listed in the basic resident register, and 5,430 participants responded (response rate: 71.3%). We conducted preventive health check-up and a total of 1,248 participants underwent a geriatric assessment (response rate:23.0%). To be eligible for the present study, individuals had to complete the gait and cognitive assessment. Ultimately, data from 1,240 community-dwelling adults aged 70 years or older were included in the analysis.

This study was conducted in accordance with the declaration of Helsinki and all participants undergoing geriatric assessment provided written informed consent under conditions approved by the Ethics Committee at Tokyo Metropolitan Institute of Gerontology.

### Gait performance measures

Gait performance was measured over a straight 5-m walkway using resistive pressure platform (P-Walk, BTS Engineering, Italy: length 5 m, width 0.7 m), which measures plantar pressure and time variables in dynamic phases [14, 15]. Participants were requested to walk at their usual pace and measured twice. We used 8 comprehensive gait parameters: gait speed (expressed as meters/second), stride length (centimeter), step length (centimeter), step width (centimeter), average foot pressure (kilopascal), maximum foot pressure (kilopascal), double support duration (millisecond), and single support duration (millisecond). All gait parameters showed coefficient of variation (CV) and we calculated mean gait parameters and CVs in each participant (excluding gait speed). To stabilize the data, we excluded the data for first step to calculate average pressure and maximum pressure. We used both stride length and step length to compare the correlation of distance between the same foot and different foot with cognitive function. The average number of steps over a straight 5-m walkway was 9±2 (minimum 2, maximum 28).

### Cognitive function

Cognitive function was assessed with the Mini-Mental State Examination (MMSE) [16], which was administrated by well-trained personnel. The MMSE has been widely used as a brief screening test for dementia and as a measure of global cognitive function; it encompasses orientation, memory, concentration, language, and praxis. The MMSE comprises 11 questions, and the score ranges from 0 to 30, with lower scores indicating poorer global cognitive ability [17,18].

### Other variables

The covariates included sex, age, years of education, body height and weight, body mass index, resting blood pressure, history of chronic disease, and chronic knee pain and backpain. Chronic disease included clinically relevant medical conditions (stroke, osteoarthrosis, disease of eye or ear, spinal canal stenosis, dementia, and depression).

### Statistical analyses

First, we calculated descriptive statistics to characterize the study population (Table 1). Second, Spearman’s rank correlation was used to compare comprehensive gait parameters with MMSE score (Table 2). Third, we used multiple regression models with Bonferroni’s method to examine independent associations between gait parameters with MMSE score, in which each gait parameters were defined as an independent variable and MMSE score was defines as a dependent variable (Table 3). In the analysis, gait parameters were used several variables that were significantly correlated with MMSE score. The covariates were sex, age, years of education, BMI, diastolic blood pressure, history of stroke, osteoarthrosis, disease of eye or ear, spinal canal stenosis, dementia, and depression, and chronic knee pain. Statistical analyses were done with SPSS (version 18.0; SPSS, Inc., Chicago, IL, USA). A *P* value of less than.05 was considered to indicate statistical significance.

**Table 1:** Baseline demographic and health characteristics of 1240 community-dwelling older adults.

Variable	Men (40.6%)	Women (59.4%)	All participants
Age (years)	77.2 ±5.0	77.2 ±4.7	77.2 ± 4.8
Years of education (years)	13.3 ±2.9	12.1 ±2.2	12.6 ± 2.6
Body height (cm)	163.3 ±5.9	150.0 ±6.4	155.4 ± 10.1
Body weight (kg)	62.6 ±8.9	51.1 ±8.1	55.8 ±10.1
BMI (kg/m^2^)	23.5 ±2.9	22.7 ±3.4	23.0 ± 3.2
Blood pressure (mm Hg)			
Systolic	144.0 ±22.0	136.8 ±19.7	139.7 ± 20.9
Diastolic	81.0 ±12.9	78.8 ±12.0	79.7 ± 12.4
History of chronic disease (%)			
Stroke	10.5	6.7	8.1
Osteoarthrosis	10.5	26.4	19.9
Disease of eye	52.4	66.0	60.5
Disease of ear	20.5	22.5	21.7
Spinal canal stenosis	17.9	17.3	17.6
Dementia	2.0	0.7	1.2
Depression	1.8	4.8	3.6
Chronic pain (%)			
Knee	23.3	34.4	29.9
Back	37.2	42.1	40.2
Gait parameters			
Gait speed (m/sec)	1.2 ±0.2	1.2 ±0.2	1.2 ± 0.2
Stride length (cm)	120.3 ±20.2	114.2 ±16.7	116.7 ± 18.4
Step length (cm)	59.8 ±10.0	56.7 ±8.3	57.9 ± 9.1
Step width (cm)	24.0 ±3.2	17.9 ±2.8	20.4 ± 4.2
Average foot pressure (kPa)	101.4 ±10.9	82.3 ±12.9	98.5 ± 11.3
Maximum foot pressure (kPa)	306.4 ±42.0	310.0 ±41.1	308.4 ± 41.4
Double support duration (ms)	66.1 ±25.5	59.7 ±25.8	62.3 ± 25.8
Single support duration (ms)	422.7 ±34.4	400.7 ±30.9	409.7 ± 34.3
Coefficient of variations for gait parameters			
CV of stride length	4.9 ±2.3	4.7 ±2.1	4.8 ± 2.2
CV of step length	7.5 ±3.5	7.4 ±3.6	7.4 ± 3.5
CV of step width	12.0 ±4.0	15.6 ±4.6	14.2 ± 4.7
CV of average foot pressure	11.8 ±4.3	12.6 ±3.9	12.3 ± 4.1
CV of maximum foot pressure	18.8 ±5.5	19.2 ±5.0	19.0 ± 5.2
CV of double support duration	33.7 ±12.9	36.6 ±12.4	35.4 ± 12.7
CV of single support duration	6.6 ±3.2	6.5 ±3.2	6.5 ± 3.2
MMSE (score)	26.9 ±2.7	27.3 ±2.5	27.1 ± 2.6

± SD. CV, coefficient of variation. BMI, body mass index. MMSE, Mini-Mental State Examination.

**Table 2:** Correlation of gait parameters with cognitive function in community-dwelling older adults.

	MMSE Score	
	Spearman’s ρ	*P*-value
Gait parameters		
Gait speed	.124	< 0.001
Stride length	.154	< 0.001
Step length	.156	< 0.001
Step width	-.100	< 0.001
Average foot pressure	.098	< 0.001
Maximum foot pressure	.042	0.139
Double support duration	-.109	< 0.001
Single support duration	.068	0.017
Coefficient of variations for gait parameters		
CV of stride length	-.141	< 0.001
CV of step length	-.145	< 0.001
CV of step width	.030	0.298
CV of average foot pressure	-.061	0.034
CV of maximum foot pressure	-.030	0.302
CV of double support duration	.054	0.061
CV of single support duration	-.085	0.003

Spearman’s rank correlation was run.

**Table 3:** Independent associations of gait parameters with cognitive function among community-dwelling older adults.

	Dependent Variable, MMSE score	
Independent Variable	Crude β (*P*-value)	Adjusted Model β (*P*-value)
Gait speed	.154 (*p*<0.001)	.080 (*p* = 0.007)
Stride length	.192 (*p*<0.001)	.123 (*p*<0.001)
Step length	.193 (*p*<0.001)	.123 (*p*<0.001)
Step width	-.101 (*p*<0.001)	-.072 (*p* = 0.078)
Average foot pressure	.108 (*p*<0.001)	.067 (*p* = 0.020)
Double support duration	-.147 (p<0.001)	-.083 (p = 0.005)
Single support duration	.096 (p<0.001)	.083 (p = 0.006)
CV of stride length	-.136 (p<0.001)	-.091 (p<0.001)
CV of step length	-.140 (p<0.001)	-.090 (p<0.001)
CV of average foot pressure	-.050 (p = 0.079)	-.041 (p = 0.138)
CV of single support duration	-.094 (*p*<0.001)	-.057 (*p* = 0.039)

Multiple regression models with Bonferroni’s method were run separately. Model adjusted for sex, age, years of education, BMI, diastolic blood pressure, history of stroke, osteoarthrosis, disease of eye or ear, spinal canal stenosis, dementia, depression, and chronic knee pain.

## Results

### Demographics

Among 1,240 participants, 59.4% were women, average (standard deviation [SD]) age was 77.2 (4.8) years, average years of education was 12.6 (2.6), average body mass index was 23.0 (3.2) kg/m^2^, average systolic and diastolic blood pressure were 139.7 (20.9) and 79.7 (12.4), 8.1% had stroke, 19.9% had osteoarthrosis, 60.5% had disease of eye, 21.7% had disease of ear, 17.6% had spinal canal stenosis, 1.2% had dementia, 3.6% had depression, 29.9% had chronic knee pain, and 40.2% had chronic backpain. The average MMSE score was 27.1 (2.6): 80.0% of participants had an MMSE score of 26 or higher and 8.1% had an MMSE score of 23 or lower. The average values of gait performance measures were as follows—gait speed: 1.2 (0.2) m/second, stride length: 116.7 (18.4) cm, step length: 57.9 (9.1) cm, step width: 20.4 (4.2) cm, foot pressure: 98.5 (11.3) kPa, maximum foot pressure: 308.4 (41.4) kPa, double support duration: 62.3 (25.8) ms, single support duration: 409.7 (34.3) ms (Table 1).

### Correlation of Gait Parameters with Cognitive Function

Correlations between gait parameters with MMSE score are shown in Table 2. Among comprehensive gait performance measures, gait speed (Spearman’s ρ = .124, *p*<0.001), stride length (ρ = .154, *p*<0.001), step length (ρ = .156, *p*<0.001), step width (ρ = -.100, *p*<0.001), average foot pressure (ρ = .098, *p*<0.001), double support duration (ρ = -.109, *p*<0.001), single support duration (ρ = .068, *p* = 0.017) for gait measures, and CV of stride length (ρ = -.141, *p*<0.001), CV of step length (ρ = -.145, *p*<0.001), CV of average foot pressure (ρ = -.061, *p* = 0.034), and CV of single support duration (ρ = -.085, *p* = 0.003) were correlated with MMSE score, respectively.

### Independent associations of gait parameters with cognitive function

The associations of gait parameters with MMSE score are shown in Table 3. After adjustment for important confounders, gait speed (β = .080, *p* = 0.007), stride length (β = .123, *p*<0.001), step length (β = .123, *p*<0.001), average foot pressure (β = .067, *p* = 0.020), double support duration (β = -.083, *p* = 0.005), single support duration (β = .083, *p* = 0.006), CV of stride length (β = -.091, *p*<0.001), and CV of step length (β = -.090, *p*<0.001) were significantly associated with MMSE score.

## Discussion

The present study of community-dwelling older Japanese is the first to show comprehensive characteristic for gait parameters of elderly with lower cognition. Older adults with lower cognitive function tend to have shorter stride length, shorter step length, shorter single support duration, lower average foot pressure, longer double support duration, higher CV of stride length, higher CV of step length, and higher CV of single support duration, in addition to slower gait speed.

We previously reported that slower gait speed and shorter step length predicted cognitive decline [10]. Verghese et al. reported pace factor such as velocity, stride length, and double support time was associated with global cognitive decline [11]. The present study confirmed those earlier results. Of interest, CV values of stride length, step length, and single support duration showed inverse association with cognitive function in this study. Martin et al. suggested that poorer cognitive function seemed to have the greatest impact on double support chase variability [9]; however, their study did not examine both single support duration and double support duration. We simultaneously assessed single and double support duration; only single support duration was significantly correlated with cognitive function. Our findings suggest that slowness of action, when body weight is carried by the left or right leg, reflects impaired cognitive processing. No previous study examined the association between CVs of single support duration and double support duration and cognitive function simultaneously; however, the present study compared these variables and identified CV of single support duration had significant association with lower cognition. Moreover, our findings were the first to show the association between average foot pressure and cognitive function. The present study suggests that lower foot pressure in which represents smaller vertical displacement of the center of gravity (COG) may reflect subjects with lower cognitive function. Namely, person with lower cognitive function tend to have unsteady gait such as erratic length and time of one step, in addition to decreasing the vertical displacement of the COG and slower speed. The characteristics for such unsteady gait might be facilitate clinical screening of high-risk persons for future dementia.

Evidence suggests that there are two mechanisms by which several gait parameters associated with lower cognitive function. First, pathological changes may indeed explain the link between unstable walk and lower cognition assessed by MMSE. The MMSE reflects the integrity of widely distributed network of cognitive domain situated in both hemispheres with left sided predominance, in addition to specific brain areas [19]. Rosano et al. reported that gait speed and double support time were associated with both white matter hyperintensities and brain infarcts [20]. On the other hand, there was a differential association of gait stride with brain infarcts, but not with white matter hyperintensities [20]. Nadkarni et al. reported the association between gait speed and total cerebellar gray matter volume [21], and Rosano et al. reported that smaller volume of prefrontal area may contribute to slower gait speed through slower information processing [22]. Rosano et al. reported that step length and double support time associated with the right sensorimotor region [23]. These pathological changes in broad brain areas may share unsteady gait and lower cognitive function. Second, functional brain inactivity may have a key role in both of gait parameters and lower cognitive function. Shimada et al. reported older adults who had high step length variability showed significant deactivations in the frontal lobe and the inferior temporal gyrus during treadmill walking by using positron emission tomography with 18F-fluorodeoxyglucose [24]. Sakurai et al. reported that slower maximum gait speed even in the range of individual difference was associated with lower normalized reginal cerebral metabolic rates of glucose in the prefrontal, posterior cingulate, and parietal cortices [25]. Such pathological changes and functional brain inactivity suggest possible mechanisms in the prodromal stage of dementia; however, the precise mechanism that controls characteristical gait parameters in this study and the regions responsible in the brain are very complex and remain to be examined in depth. Further research is needed to investigate the precise mechanism that determines specific gait measures associated lower cognitive function.

This study has several strengths. First, our relatively large sample size of community-dwelling participants enabled multiple regression models to examine independent associations between gait parameters with MMSE score using comprehensive covariates, such as demographics, health characteristics, and chronic disease. Second, gait performance measures in the present study include unique variables by using resistive pressure platform, for example, average and maximum foot pressure, and CVs of gait measures. Our cross-sectional study showed the characteristics with lower cognitive function, however, further longitudinal study might conduct new insights regarding physiological mechanism in the prodromal stage of dementia.

Several limitations of this study warrant mention. First, because of the healthy volunteer effect, the participants were healthier than the general elderly population. Although we had mailed all residents in Takashimadaira, Itabashi ward, Tokyo aged 70 years or older who was listed in the basic resident register, 1,248 of 5,430 (response rate:23.0%) participated in our study and remaining those in the poor health would be excluded from the study. Therefore, the average values of gait performance measures may be better than general population’s (The average values of gait speed in the present study: 1.2 meters/second). However, the present participants are representative of persons who take part in community-based health checkups, and the characteristic for gait parameters of elderly with lower cognition in this study would thus be applicable to such persons. Second, chronic disease was determined by self-reported clinically relevant medical conditions. Previous studies used medical record data to assess chronic disease [1, 11]. This limitation was not available in the present study, and this limitation might have led to some misclassification. Third, a previous study recommended that older adults and people with Parkinson’s disease needed to walk more than 30 steps for researchers to calculate levels of gait variability accurately [26]. Although we measured gait parameters twice over a straight 5-m walkway, our setting was shorter than this recommendation (the average number of steps at one time was 9). Future studies should measure with a longer walkway for these measurements. Finally, we assessed cognition using the MMSE. Although, the MMSE is a convenient measure of global cognition, it is not the only instrument for such assessment. Because the present study did not include a comprehensive cognitive battery, further investigation is needed in order to identify associations of gait performance measures with other measures of cognitive function.

In conclusion, older adults with lower cognitive function tend to have shorter stride length, shorter step length, shorter single support duration, lower average foot pressure, longer double support duration, higher CV of stride length, higher CV of step length, and higher CV of single support duration, in addition to slower gait speed. Our findings indicate that older persons with lower cognitive function tend to show unsteady gait, such as erratic length and time of one step, in addition to decreasing the vertical displacement of the center of gravity and slower speed.
